# Determinants of Portion Size in Children and Adolescents: Insights from the UK National Diet and Nutrition Survey Rolling Programme (2008–2016)

**DOI:** 10.3390/nu11122957

**Published:** 2019-12-04

**Authors:** Pam Blundell-Birtill, Marion M. Hetherington

**Affiliations:** School of Psychology, University of Leeds, Leeds LS2 9JT, UK; m.hetherington@leeds.ac.uk

**Keywords:** portion size, snack food, children, adolescent, chocolate, crisps, high energy density food, NDNS

## Abstract

Large portion sizes have been identified as contributing to overweight and obesity, particularly in children and adolescents. This study examined predictors of portion sizes of high energy snack foods eaten by children aged 1.5–18 years. Specifically, we examined whether portion sizes were adjusted for age, and what external features of the environment might be linked to large portion sizes. Portion sizes were derived from four-day food diaries that form the UK National Diet and Nutrition Survey. Diaries from 5942 children were examined and multilevel models were used to discover whether age, gender, location, time of day, household income, and watching TV while eating predicted portion sizes of savoury snacks, chocolate, confectionery and biscuits. Portion sizes of all the target foods were predicted by age. Boys had larger portions, and portion sizes were larger when target foods were consumed later in the day. Portion sizes were larger outside the home, for example in leisure venues, but the target foods were eaten more frequently in the home. As dietary patterns change to include more snack intake outside the home, these locations could be an important space to target for interventions for portion control.

## 1. Introduction

The World Health Organization has proposed that limiting portion sizes to reduce overall energy intake could lead to a reduced risk of unhealthy weight gain [1]. In particular, large portion sizes of beverages, meals and snacks were identified as a potential determinant of excess intake, in turn contributing to overweight and obesity.

Portion sizes of high energy dense foods (HED) have increased across Westernized nations [2,3,4,5]; and this may set the social norms for portion sizes of these foods. Since early diet and eating patterns in childhood are known to shape eating habits in later life [6], it is imperative to understand what portion sizes are offered to children as they navigate the external food environment. It is also useful to note features of the external food environment which predict exposure to large portion sizes of palatable, energy dense foods.

The portion size effect (PSE), whereby intake is increased by offering large portions, has been demonstrated in a number of laboratory-based experiments [7,8]. In these studies, large portion sizes of palatable, energy dense foods produce a reliable increase in energy intake compared to small or regular portion sizes in children and adults (see [9] for a review). The PSE is especially strong when portion sizes are large and foods are energy dense, a phenomenon labelled “double trouble” [10]. Providing large portion sizes of energy dense and nutrient poor foods has been identified as a risk factor for overweight in very young children [11].

Parents are generally in control of what is served to young children. Johnson et al., [12] measured amounts offered and consumed at three meals by children and parents at home. They found that amounts served to children explained 73% of the variance in children’s intake and large portions resulted in large intakes. Additionally, the amount of food parents served to themselves strongly correlated with the amounts served to their child [12]. Therefore, parents may set an expectation of what is appropriate to eat at a meal by example. Additionally, parental portion size choices better predict a child’s BMI than children’s portion size choices [13]. If children rely on external cues, such as served portion sizes, to determine how much food to consume, then they may learn to rely less on internal cues of satiation and satiety [8]. Additionally parents learn to provide the food portions that are appropriate for their child [14]. However, parents do not necessarily know what portions are age appropriate [15].

The energy density (kcal/g) of foods is a significant contributor to the PSE since foods high in fat, sugar and salt are most often the items which are overconsumed when offered in large portions. For example, Albar et al. [16] defined HED items as those containing more than 2.5 kcal/g and reported a positive relationship between total energy intake and body mass index (BMI) in adolescents. In this study, the portion sizes of a number of HED foods (biscuits, cheese, cream and cakes) were found to be positively associated with BMI.

Provision of large portions of HED foods is implicated in excess intake and so reducing the portion sizes of these foods (known as downsizing) may help to limit intake [17]. Indeed Public Health England (PHE, 2018) [18] has launched a campaign to encourage parents to provide small, 100 kcal snacks (no more than two per day) and manufacturers have responded to this by developing snack ranges which meet this suggestion. Therefore, the portion sizes of highly palatable HED foods consumed by children and adolescents should be characterized in relation to this guidance. We need to understand whether portion sizes offered by parents are appropriate for their child’s age, and what factors contribute to large portion sizes of these foods. For example, it is expected that portions of HED items will be smaller for young children as they have lower energy needs than adults [19]. Also, it is expected that portion sizes of HED items will be influenced by context such as eating outside the home, eating while watching TV and eating in groups. It is known that eating at food outlets and ‘on-the-go’ is associated with intake of HED items [20] in adults, and that eating while distracted such as eating while watching TV is known to promote intake in adults [21]. Additionally, boys have higher energy needs than girls [19] and so might be expected to have larger portions, while girls tend to be more diet conscious, particularly as they get older [22,23], and so there may be interactions between gender and portion size for HED foods.

Therefore, this study examined the portion sizes reported of four different types of HED, non-core target foods in children aged 1.5 to 18 years, by conducting a secondary analysis of data in the UK National Diet and Nutrition Survey (NDNS). This is a nationally representative data set of four-day food diaries collected between 2008–2016. Toumpakari et al. [24] identified “crisps and savoury snacks, chocolate and biscuits” as among the top five non-core foods eaten by adolescents. Furthermore, the effects of age, gender, place, time of day, and whether the food was eaten in front of the TV were examined. It was predicted that smaller portions of foods would be eaten by younger children and that context would influence the portion size of HED foods consumed.

## 2. Materials and Methods

### 2.1. Sample

Secondary data analysis was conducted on data collected between 2008 and 2016 as part of the UK National Diet and Nutrition Survey (NDNS) years 1–8 [25]. The NDNS is a continuous, cross-sectional survey that collects detailed information on food consumption of the general population (around 1000 per year) aged 1.5 years and over living in private households in the UK. The survey aims to collect data from 500 adults (aged 19+ years) and 500 children (aged 1.5–18 years) per year, with numbers boosted to 400 participants per year from Scotland (years 1–4 only) and 200 per year from Wales and Northern Ireland (years 1–8). Field work is conducted throughout the year to address potential seasonal variations in food consumption. The full details of the NDNS survey design and sampling methods are available elsewhere [25]. In brief, a random sample is drawn from the Postcode Address File, which is then clustered into small geographical areas (Primary Sampling Unit; PSUs) randomly selected from across the UK. From each PSU, 27 addresses (28 in Years 6–8) were randomly selected. Where more than one household was present at an address, the interviewer selected the household to be interviewed at random. As the survey aimed to have an equal number of adults and children, at some addresses only children were selected to take part. Data for all children aged < 19 years were included in this analysis.

### 2.2. Dietary Data

Within the NDNS, assessment of portion sizes was carried out using four-day estimated food diaries. Only participants who completed three or more diary days were included (106, 1.8% completed only three-day diaries, and 5836, 98.2% completed four-day diaries). There were two main parts to the survey, namely an interviewer stage and a nurse visit. Only data from the interviewer stage have been considered in this study. During the interview stage participants were given an extensive face-to-face computer assisted personal interview, a four-day food diary and self-completion questionnaires. For the food diaries participants were asked to record everything they ate and drank over four days, along with the time, where they were, and who they were with. They were asked to record the eating as it happened, rather than from memory. Participants were asked to save all wrappers and packaging. They were asked to estimate portion sizes using household measures such as teaspoons, slices, cups and tablespoons, as well as using weights from labels, number of items, and picture examples provided in the documentation. Participants were asked to record only the amount that was actually eaten, that is, to take leftovers into account. For children aged < 12 years, a parent or carer was asked to complete the four-day diary with help from the child as appropriate. Children aged ≥ 12 years were asked to complete the diary themselves. A second brief visit or phone call was included during the diary period to ensure compliance and allow the participants to ask any questions. Following completion of the food diaries participants were visited a final time, to collect the diary and review and edit possible omissions.

Diaries were coded by trained coders and editors in the NDNS research team. Food intakes were entered into a modified version of the Diet in Nutrients Out system [26], and food composition data from the Department of Health NDNS nutrient databank was used. To determine portion size, coders could select household measures within the Diet in Nutrients Out system, or enter portion sizes in grams where this was recorded or available from packaging.

This study has focused on high energy density foods that are generally eaten as snacks. Data were selected from the full NDNS data set where both the main food group description and the recipe main subgroup was either chocolate confectionery, crisps and savoury snacks, biscuits, or sugar confectionery. This meant that for example, chocolate contained within a yoghurt was not counted as chocolate. Within the NDNS data set, portion sizes were provided in grams from diary entries. These were then converted into energy, and nutrient intakes by the NDNS project team.

### 2.3. Other Variables

Within the NDNS data set the gender of participants was recorded, along with age in full years. Exact age is not provided. When completing diet diaries participants were asked to include the time of eating, and whether they were watching TV. Where it was not specified that people were watching TV, we have classified it as “not watching TV” (15.5% of occasions). The z-score equalized household income was included (equalized income is a measure of household income that takes account of the differences in a household’s size and composition), as was the BMI category. Within the NDNS dataset, BMI is calculated from height and weight data recorded by the interviewer. Children are then categorized within a range of weight categories from having a healthy weight (which includes underweight) to having overweight or obesity, using the BMI WHO cut-offs (85th/95th centile for 2–3-year olds (inclusive) and UK90 for 4–18-year olds. The 582 children for whom data was not made available were classified as having a healthy weight. 

A new categorical variable for ‘where foods were eaten’ was created from responses in the original NDNS survey [20]. Places were classified as ‘On the go’-on public transport, on the street, and outside; “at home” included all possible places within the home; “at school” included all locations at school, and also included all work locations (this only applied to a small number of the older children in the sample); leisure included community and day centers, leisure activities, shopping, tourist attractions, cinema, places of interest, public hall or function rooms, and sports club and sports leisure venues; food outlets included coffee shops, cafes, fast food outlets, pubs and restaurants. All other places were categorized as ‘other’-including homes of carers, friends or relatives, holiday accommodation and places of worship.

### 2.4. Data Analysis

Following the methodology of Ziauddeen et al. [20], eating occasions that were within 15 min of each other, in the same place, and with the same people, were combined into single eating occasions. In order to calculate the portion size (in grams) that formed the outcome variable, the portion sizes of each of the target foods at that occasion were then summed. For example, if a child ate 5 g of crisps and 10 g of another savoury snack, these would be combined to be a portion size of 15 g. If they ate half a packet of crisps and half a chocolate bar these would be considered separate portions as they are different food categories.

The NDNS data sets for years 1–4 (2008/09–2011/12), years 5–6 (2011/12–2013/14) and years 7–8 (2014/15–2015/16) were combined. The NDNS requires weights to adjust for differences in sample selection and response, to adjust for differential selection probabilities of individuals and non-responses of individuals. Survey weights were calculated for the whole of the <19 year old sample according to the instructions of the NDNS [25] and these weights were then used throughout the rest of the analysis. Age was centered to allow for simple interpretation of the model intercept coefficient (the intercept gives the baseline portion size in grams), but not scaled.

In order to examine associations between the frequency of consumption and where, and when the different target foods were consumed, chi-squared tests were conducted on the total number of each target food that was consumed in each place. Then to investigate the frequency with which each target food was consumed, linear models were created to predict the frequency of each target food, each day, in each place. The predictors investigated were location, age and gender of the child. Multilevel models were not used in this analysis, as they produced singular solutions as many children consumed each target food only in one specific location. We have made no adjustment for whether diaries were for three or four days, as the number of three-day diaries was so small, and we are looking at frequency per day. Therefore, the number of full days per child would have had little effect on the data.

Portion size data for each target food type was explored separately. The data were analyzed using multilevel models, with the individual as a random factor, and the predictors of interest as fixed factors. Multilevel models deal well with missing data, and so the fact that we only have three days of data for some of the children been accounted for within the model. In the first model, all predictors were entered. Interactions between age, sex and the other predictors were then added. Where these improved the model, as assessed by analysis of variance, these interactions were retained. Analysis of variance with Satterthwaite approximation for degrees of freedom were used to assess the significance of individual predictors. Alpha of 0.01 was adopted for decisions involving model building. The portion size of the target food in grams on each eating occasion was the outcome variable.

In order to investigate the contribution of these target, non-core foods to the total diet, we extracted the total amount of energy consumed per day per child from the NDNS. We then summed the total energy from all four target foods across each day, for each child, and calculated the percentage of energy that came from these foods per day. We then used a multilevel analysis to investigate whether the percentage of energy from the target foods varied with age, gender and their interaction. We also examined whether BMI category and z-score equivalized income, and their interactions with sex and age, added to the models.

Data analyses were conducted using RStudio 1.1.383, with R (version 3.5.2, Eggshell Igloo), lme4 1.1.20 lmerTest 3.1.0 tidyverse 1.2.1 foreign 0.8.71.

## 3. Results

Across the four days of diet records there were 478,773 food entries, for 5942 children aged 1.5–18 years old. Of these 4375 children consumed biscuits 12,455 times, 3426 children consumed chocolate on 7254 occasions, 2279 children ate confectionery on 4336 occasions and 4063 children ate crisps and savoury snacks 9279 times.

### 3.1. Frequency of Consumption of Target Foods

Table 1 shows the frequency (with percentage of the total) each target food was eaten in each location, at different time points, and whether the target food was eaten in front of the TV or not. The majority of the target snacks were eaten in the home, between 14:00 and 17:00. Approximately one third of the foods were eaten in front of the TV. Sugar confectionery was consumed the least frequently, and biscuits were most usually consumed.

Chi-squared tests were conducted to test the associations between where the target foods were consumed and what was consumed. These showed a significant association (χ^2^ (15) = 1374, *p* < 0.001). Examination of the residuals demonstrated that chocolate and confectionery were less likely than expected to be eaten at school, while biscuits were less likely than expected to be eaten on the go. Confectionery was more likely to be eaten in a leisure setting and on-the-go, while crisps and savoury snacks, and biscuits were more likely to be eaten at school. Similarly, a chi-squared test demonstrated there was an association between watching TV and eating specific target foods (χ^2^ (3) = 46.5, *p* < 0.001). Children were more likely to eat chocolate while watching TV, and less likely to eat confectionery while watching TV. Finally, time period was also significantly associated with target food (χ^2^ (18) = 1500, *p* < 0.0001), such that children were less likely to eat crisps and savoury snacks in the morning, and most likely to eat them at lunch time. Children were less likely to eat chocolate and confectionery at lunch, and more likely to eat biscuits in the morning at breakfast.

In order to examine what predicted the frequency of consumption of each of these target foods in a day, the number of times a target food was consumed per day, per location and per child was calculated. Table 2 shows the calculated parameters. In all cases, the meal location was a significant predictor of frequency of consumption. The home was the place where all target foods were eaten most frequently, followed by school. Age was only a significant predictor of frequency for crisps and savoury snacks (with older children eating them more often), although the effect size here was very small, and sex was only a significant predictor for confectionery, with girls eating confectionery more frequently than boys.

### 3.2. Portion Sizes of Target Foods

**Figure 1:**
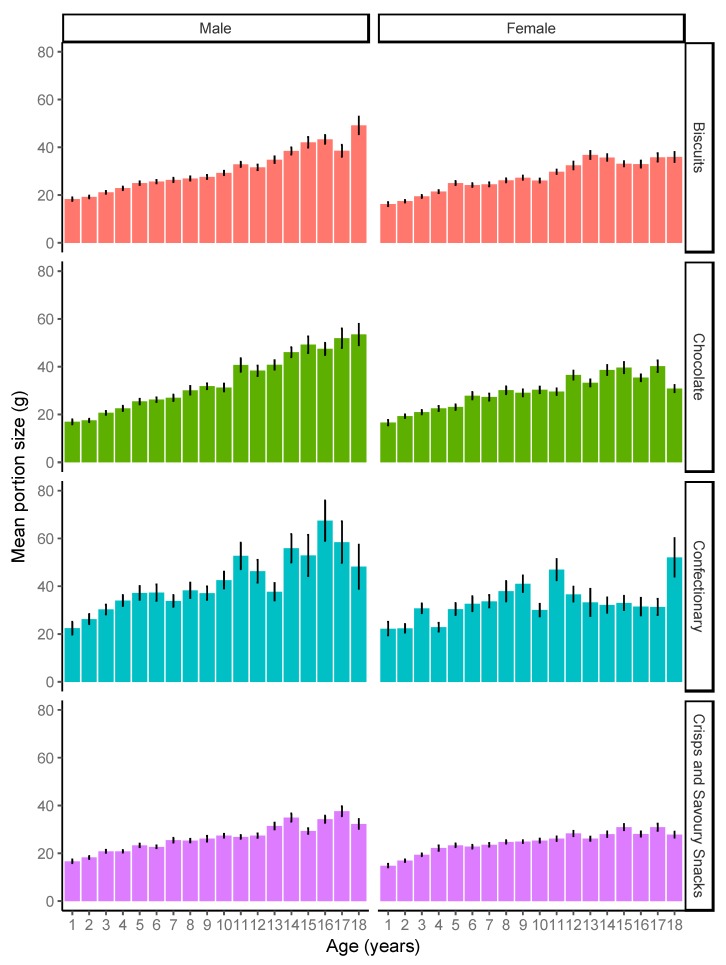
Mean portion size (g) for each of the target foods, by child age for males and females. Error bars indicate standard error.

**Figure 2:**
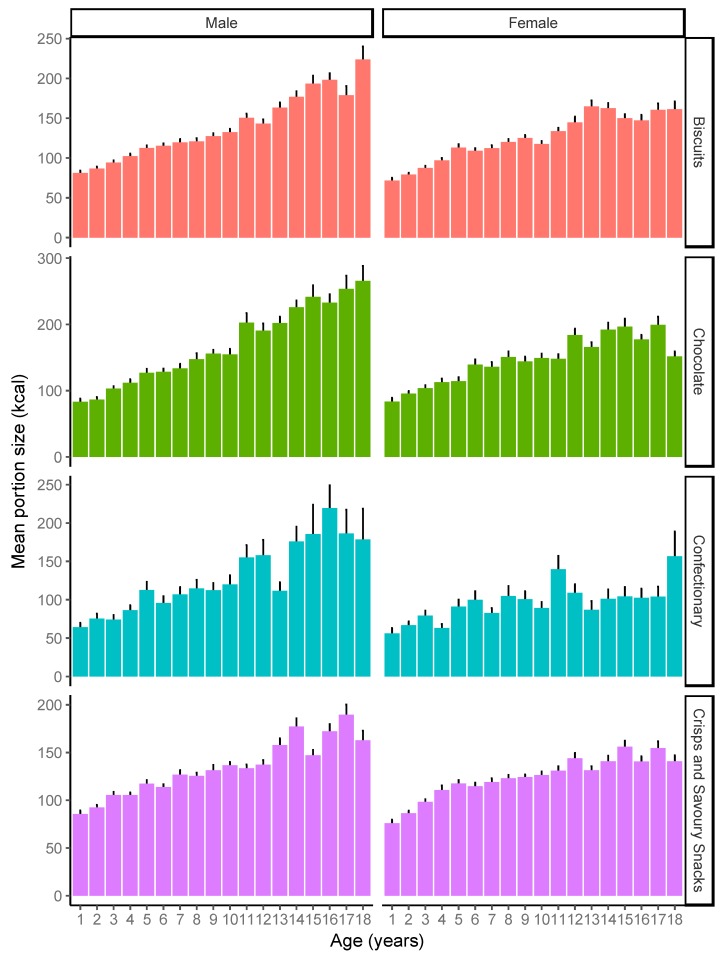
Mean portion size (kcal) for each of the target food types, by child age for males and females. Error bars indicate standard error.

Figure 1 shows portion sizes by weight (grams) for each target food by child age, and separately for girls and boys. As can be seen, generally portion sizes increased with age, but generally this effect was more pronounced for boys than for girls, possibly suggesting that older girls will not increase their portion size past a certain point. The effect of age varied by food, with a smaller gradient for crisps and savoury snacks than for the other foods. Figure 2 shows the equivalent data for portion sizes by energy content (kcal). Energy intake follows much the same pattern as for weight.

### 3.3. Predictors of Portion Sizes (g) of Target Foods

The interactions that were retained in the models varied by target food. For biscuits, the interactions between age and location, sex and time of eating, age and BMI category, and the three-way interaction between age, sex and BMI category all improved the model and so were retained in the final model. For chocolate, age, sex, location, time of day, watching TV and income were all significant predictors of portion size, as were the interactions between age and sex, and sex and time of day. For crisps and savoury snacks, only the interactions between age, sex and location of eating, and between age and time of day improved the model, and so were retained in the analysis, whereas for confectionery, only the interactions between age and location and age and sex added to the model and were kept. The final models are presented in Table 3. In all the models the baseline level of intake, for the average aged child, was between 25–30 g.

For all of the target foods, portion size increased with age and portions were smaller for girls than for boys. The interactions observed varied between the target foods. For biscuits, the effect of age was larger in food outlets and in boys classed as having overweight. For both chocolate and confectionery foods, the effect of age was smaller for girls than for boys. For the category crisps and savoury snacks, there was a larger effect of age at leisure venues, although the three-way interaction demonstrated that this effect was smaller for girls. Additionally, there was a smaller effect of age on portion sizes of crisps and savoury snacks at food outlets.

Portion sizes of chocolate, crisps and savoury snacks and confectionery were larger in leisure venues, and for confectionery this effect increased with age. Portion sizes of confectionery were also smaller in school locations than home. Portion sizes were larger in leisure locations, and this effect increased with age. For crisps and savoury snacks, the effect of leisure venues was smaller in girls. Portion sizes of crisps and chocolate were larger when ‘on the go’, than at home, and this effect also increased with age. Portion sizes of biscuits, chocolate, crisps and savoury snacks were larger when eaten in front of the TV.

For biscuits, portion sizes were larger during the time interval noon to 13:59, although this effect was smaller for girls. Portions of chocolate were largest between 09:00 and 10:59., and between 20:00 to 21:59 compared to the rest of the day whereas portion sizes of confectionery were larger in the afternoon. People with larger household equalized incomes had smaller portions of chocolate and biscuits.

### 3.4. Energy Consumption Due to Target Foods

In order to examine where the most energy from each of these food sources was consumed, the kcal per day per place per child was calculated. Linear models were then calculated to investigate the effects of age, sex and meal location on energy intake per day at each location. Summary parameters for these models are shown in Table 4. In all models, age, sex and meal location were highly significant predictors (all *p* < 0.001). These demonstrate that, despite the differences in portion sizes and frequency described above, most energy from the target foods is consumed in the home. The effect of age was small, such that for example, per day, a four-year old would consume 10 kcal less crisps and savoury snacks than a 14-year-old.

### 3.5. Contribution of Energy Consumption Due to Target Foods to the Overall Diet

In order to examine the contribution of energy from the target foods to the overall diet, a multilevel model was created with percentage of daily energy from the target foods as the outcome variable. The results of this analysis can be seen in Table 5. In the model, age, sex, income and the interactions between age and sex, and sex and income were all significant predictors of the percentage of energy that came from these target foods. While these effects were significant, they were small, such that a 14-year-old would have 1.6% more of their energy from these target foods than a 4 year old. Additionally, the effect of gender was such that boys get 0.5% more of their energy from these foods. The interaction with equivalized income shows that boys from households with lower equivalized income obtained more of their daily energy from these target foods, but this effect was smaller for girls.

## 4. Discussion

Overall, portion size varied by food type, age, sex, and meal location. The frequency of consumption varied by place, and most snacks (weight and energy) were consumed in the home. In terms of energy from snacks, for children over five, most of the portions were larger than the PHE recommended 100 kcal.

For all target foods, age was an important predictor of portion size, indicating that downsizing is done in accordance with age. However, the effect of age was greater for confectionery and chocolate, than for crisps and savoury snacks, and biscuits. This accords with the findings of Wrieden et al. [27] who used both NDNS and weighted food diaries to estimate portion sizes of 75 different foods and found larger differences in portion sizes between the youngest and the oldest age groups for chocolates than for crisps and savoury snacks. This may be explained by a tendency for some foods to be given in packages of a certain size (e.g., 25 g for crisps and savoury snacks) or counted as units (one or two biscuits). Indeed Eck et al., 2018 [28] report that parents use pre-packaged food to facilitate portion control. Similar strategies have been reported in the United States [29]. While the portion size effect has been shown to occur across food types for both units and amorphous foods (see Reale et al. [30]), in this large, nationally representative dataset, packaging and units appear to be a significant influence on portion size for certain foods such as crisps and savoury snacks and biscuits. Whereas for chocolate and confectionery, which are easy to segment and available in a wider range of sizes, the amounts consumed by children varied much more with age. This accords with an experimental study by Vandenbroele et al. (2019) [31] which reported that the size of food units was more important in determining portion size than the number of food units.

Sex was also a significant predictor of portion size, and again this was a larger factor for chocolate and confectionery than for biscuits and crisps and savoury snacks. For girls, portion sizes of chocolate and confectionery were smaller by around 8 g for chocolate and confectionery, but only around 1.5 g for crisps and savoury snacks and biscuits. The significant interaction between age and sex for chocolate and confectionery with a negative parameter demonstrates that the effect of age is smaller in girls for these foods. This suggests that efforts to downsize portions of chocolate and confectionery could be targeted at older boys in particular. This finding is consistent with research across both Europe and the United States demonstrating that boys consume more sugar and sweets, and generally have less nutrient dense diets [32]. Additionally research in Australia [33] reports that men eat larger portions of several foods including chocolate, suggesting that this difference may track into adulthood.

Location was a significant influence on portion size, with larger portions of chocolate, confectionery and crisps and savoury snacks being consumed in leisure locations. Again, these effects were often dependent on age and sex, with effects being smaller in girls and being larger with age. While larger portions were consumed in these locations, they were not eaten very frequently. The foods studied were consumed more frequently, and with greater energy from these foods, in the home. Children report difficulty in avoiding overeating highly palatable foods in restaurants and when others are overeating [28]. The findings that portion sizes were larger when eaten outside the home than inside are consistent with existing findings in both adults and children [24,34]. In adults, eating meals outside of the home is associated with an overall larger weekly energy consumption, this has not been found in children [35]. Our analysis suggests that portions of specific snack foods are larger outside the home and a potentially important target for downsizing.

In this analysis, watching TV only contributed to portion size of biscuits. Household income was only important for biscuits and chocolate, with those on lower incomes having larger portions. These effects were independent of age and sex. Portion sizes of foods tended to be larger later in the day, with biscuits and crisp portions being larger around lunchtime. BMI category was only a predictor of portion size for biscuits. The lack of effect of BMI on portion sizes is consistent with other research (e.g., [36]) which does not find a difference in snack food intake between adolescents with and without overweight/obesity. However, these groups may be more susceptible to misreporting, which this analysis has not taken into account [16]. However, generally, under-reporting increases with age [37] and so while effects of BMI may be underestimated, so too will effects of age, which are the principle points of interest of this study. Girls show more underreporting which may account for some of the differences between sexes [38].

Portion sizes of chocolate were larger during times that could be considered snack times (between meals), while portion sizes of confectionery increased throughout the day.

The proportion of energy from these target non-core foods was around about 15%, which is lower than typically reported (e.g., [24,39]), but this reflects that this study does not include all non-core foods, instead choosing to examine the factors that affect portion size of specific types of food.

However, an additional finding from the energy intake data was that children from lower income families consumed more of their total energy intake from the target foods than children from higher income families, and this was greater for boys than for girls. This finding may be attributable to the greater accessibility, convenience and affordability of the target foods for lower income families (see [40]) and if maintained over time could contribute to the social gradient in adult obesity [41]. While this statistical analysis can tell us about general trends, examination of the data files illustrated some of the ways in which the foods were consumed and downsizing was achieved. For example, a child would consume at the same time fractions of several chocolate bars, suggesting that these were being shared between siblings or friends. In an examination of the types of foods eaten, younger children were more likely to eat small, single unit packaged chocolate bars while older children would eat chocolate that came as a larger single unit—Indicating the way that packaging, and age appropriateness of foods, can assist in portion control. However, the present research suggests that the smaller snacks, which meet the PHE guideline of being under 100 kcal, will need to be marketed in a way that makes them appeal to older children and adults, otherwise they will only be effective for younger children.

This study only considers portion sizes and frequency of eating four target non-core foods. However, similar patterns are found across the four target foods, suggesting the findings should generalize to other non-core foods. This study only considers findings from the UK. As Kirwin et al. (2016) [42] report, portion sizes vary considerably across Europe, suggesting that the findings of this study may not generalize to other countries. In this study we have not considered how findings may change across the years of the NDNS, as we were principally concerned with whether downsizing occurs with age, however evidence suggests that there is little change in diet from years 1–2 of the NDNS and years 5–6 [43].

In conclusion, this research provides evidence that portion sizes of palatable, high energy density snack foods are adjusted according to age of the child, and differ by food type, sex and location. Overall, smaller portions of each food were eaten by younger children and these increased with age. Boys consumed larger portions and showed bigger effects of age on portion size; and portions were significantly larger when eaten in leisure venues.

Age-sensitive downsizing occurs for these non-core foods. Therefore attempts to reduce energy intake in children by limiting these foods may be better focused on reducing the frequency of consumption in the home, perhaps by replacing with a low energy density snack [44] as well as reducing the size of servings of these foods when outside the home. These data suggest that future interventions aiming to improve portion size control should be tackled in out of home contexts, especially leisure venues. However, reducing the frequency of consumption in the home is especially important given the dominance of home based intake for these foods [45]. Downsizing efforts using child sized portions and packaging may assist parents in meeting guidance to provide 100 kcal snacks [18] and the present study suggests this is achievable.

## Figures and Tables

**Table 1:** Total number of occasions (and percentage of the total) that each of the target foods was consumed in each location, during each time period, and whether they were eaten in front of the television.

Where	Biscuits		Chocolate		Confectionery		Crisps & Savoury Snacks	
**Home**	6831	54.8%	4114	56.7%	2114	48.8%	4553	49.1%
**School (incl. work)**	3151	25.3%	1026	14.1%	495	11.4%	2360	24.4%
**Other**	1170	9.4%	743	10.2%	467	10.8%	900	9.7%
**On the go**	802	6.4%	909	12.5%	815	18.8%	986	10.6%
**Leisure**	308	2.5%	299	4.1%	318	7.3%	325	3.5%
**Food Outlet**	193	1.5%	163	2.2%	127	2.9%	155	1.7%
**Watching TV**								
**No**	6747	54.2%	3765	51.9%	2408	55.5%	4940	53.2%
**Not specified**	1793	14.4%	1002	13.8%	702	16.2%	1364	14.7%
**Yes**	3915	31.4%	2487	34.3%	1226	28.3%	2975	32.1%
**Time of day**								
**06:00 to 08.59**	704	5.7%	239	3.3%	90	2.1%	81	0.9%
**09:00 to 11.59**	2486	20.0%	971	13.4%	612	14.1%	1547	16.7%
**12:00 to 13.59**	2949	23.7%	1382	19.1%	748	17.3%	3191	34.4%
**14:00 to 16.59**	3143	25.2%	2126	29.3%	1460	33.7%	2262	24.4%
**17:00 to 19.59**	1832	14.7%	1523	21.0%	917	21.1%	1195	12.9%
**20:00 to 21.59**	1086	8.7%	843	11.6%	422	9.7%	794	8.6%
**22:00 to 05.59**	255	2.0%	170	2.3%	87	2.0%	209	2.3%

**Table 2:** Parameters for linear models predicting frequency of consumption of the target foods.

Factor/Predictor	Biscuits	Chocolates	Confectionery	Crisps and Savoury Snacks
Intercept	0.72	0.67	0.58	0.55
Age	0	0	0.001	0.001
Sex (reference category male)	0.002	0.002	0.013	0
Meal location (reference category home)				
Leisure	−0.7	−0.6	−0.5	−0.5
Food outlet	−0.7	−0.6	−0.6	−0.5
On the go	−0.6	−0.5	−0.4	−0.4
School	−0.4	−0.5	−0.4	−0.3
Other	−0.6	−0.6	−0.5	−0.4

**Table 3:** Results of analysis of variance by Satterthwaite’s method, and parameters from multilevel modelling for portion sizes of all the target foods.

	Biscuits		Chocolate		Confectionery		Crisps and Savoury Snacks	
Factor/Predictor	F-Test	Parameter	F-Test	Parameter	F-Test	Parameter	F-Test	Parameter
Intercept		27.8 ***		26.9 ***		28.9 ***		26.1 ***
Age	F(1, 6876) = 290.3 ***	1.17 ***	F(1, 3131.1) = 433.1 ***	2.3 ***	F(1, 3177) = 68.3 ***	2.2 ***	F(1, 9164.8) = 148.2 ***	1.5 ***
Sex (reference category male)	F(1, 6706) = 5.7 *	1.5	F(1, 4662.4) = 35.6 ***	−8.9 **	F(1, 2076) = 26.1 ***	−8.5 ***	F(1, 7430.5) = 24.5 ***	−1.9 **
Meal location (reference category home)	F(5, 12.80) = 4.2 ***		F(5, 7239.3) = 10.4 ***		F(5, 4195) = 35.0 ***		F(5, 9004.5) = 16.1 ***	
Leisure		2.7 *		7.7 ***		28.4 ***		11.4 ***
Food outlet		0.9		−1.9		4.8		3.3
On the go		1.2		4.7 ***		1.1		3.1 ***
School		−1.2 *		−1.6		−6.7 **		0.2
Other		1.4 *		0.7		−2.6		0.8
Time of meal (reference category 06:00 to 08:59)	F(6, 11740) = 2.9 **		F(6, 7093.5) = 5.5 ***		F(6, 3947) = 5.1 ***		F(6, 8656.6) = 4.7***	
09:00 to 11:59		2.3		7.0 **		−8.5		−1.6
12:00 to 13:59		3.7 **		2.6		12.1 **		−1.6
14:00 to 16:59		2.1		5.9 *		12.7 ***		−1.0
17:00 to 19:59		3.0 *		5.5 *		16.4 ***		0.4
20:00 to 21:59		1.4		8.8 ***		14.7 ***		1.9
22:00 to 05:59		2.2		6.4		14.6 **		−4.0
Watching TV	F(1, 12077) = 22.5 ***	2.2 ***	F(1, 7219.6) = 5.3 *	1.7 *			F(1, 8909.5) = 19.7 ***	2.0 ***
Income	F(1, 4097) = 11.7 ***	−1.0 ***	F(1, 2674.3) = 22.5 ***	−1.9 ***			F(1, 3849.7) = 5.6 *	−0.6
Age x Sex			F(1, 3043.2) = 41.2 ***	−1.1 ***	F(1, 2133) = 16.8 ***	−1.5 ***	F(1, 7388.1) = 8.3 **	−0.2 *
Age x Meal location	F(5, 12235) = 3.5 **				F(5, 4281) = 7.3 ***		F(5, 9142.3) = 15.0 ***	
Age—Leisure		0.4				3.2 ***		2.7 ***
Age—Food outlet		0.9 **				−0.5		−1.1 **
Age—On the go		0.3				0.1		0.6 ***
Age—School		0				0.2		−0.1
Age—Other		0.3 *				−0.2		0.2
Age x Time of meal							F(6, 8747.9) = 3.0 **	
Age—09:00 to 11:59								−0.7
Age—12:00 to 13:59								−0.6
Age—14:00 to 16:59								−0.6
Age—17:00 to 19:59								−0.2
Age—20:00 to 21:59								−0.7
Age—22:00 to 05:59								0.1
Sex x Meal location							F(5, 9021.3) = 3.2 **	
Female—Leisure								−7.5 ***
Female—Food outlet								−2.3
Female—On the go								0.4
Female—School								−0.3
Female—Other								−0.5
Sex x time of meal	F(6, 11745) = 2.5 *		F(6, 7104.5) = 3.0 **					
Sex—09:00 to 11:59		−3.8 *		1.9				
Sex—12:00 to 13:59		−4.5 **		8.0 *				
Sex—14:00 to 16:59		−4.6 **		4.2				
Sex—17:00 to 19:59		−5.6 **		5.5				
Sex—20:00 to 21:59		−4.8 *		0.9				
Sex—22:00 to 05:59		0.9		−1.6				
Age x BMI category	F(2, 4463) = 7.6 ***							
Age—Overweight		1.0 ***						
Age—Obesity		0.2						
Age x Sex x Meal location							F(5, 9132.3) = 6.6 ***	
Female—Age—Leisure								−2.1 ***
Female—Age—Food outlet								1.1 *
Female—Age—On the go								−0.3
Female—Age—School								0.1
Female—Age—Other								0.1
Age x Sex x BMI category	F(2, 4453) = 3.8 *							
Age—Sex—Overweight		−0.9 *						
Age—Sex—Obesity		−0.5						

*** *p* < 0.001, ** *p* < 0.01, * *p* < 0.05.

**Table 4:** Parameters for linear models predicting daily kcal of consumption of the target foods.

Factor/Predictor	Biscuit	Chocolate	Confectionery	Crisps and Savoury Snacks
Intercept	95	103.5	61.6	74.5
Age	1.3	1.7	1.1	1
Sex (reference category male)	−2.4	−3.7	−4.3	−2.1
Meal location (reference category home)				
Leisure	−89.6	−93.6	−40.2	−68.0
Food outlet	−90.8	−98.8	−56.1	−71.7
On the go	−82.9	−77.2	−34.5	−57.5
School	−51.8	−74.4	−47.3	−38.7
Other	−78.3	−83.1	−47.0	−60.2

**Table 5:** Results from analysis of variance with Satterthwaite’s method, and parameters for multilevel models examining percent contribution of the four target foods to daily energy consumption.

Factor/Predictor	F-Test	Parameter
Intercept		15.64 ***
Age	F(1, 5805) = 94.7 ***	0.16 ***
Sex (reference category male)	F(1, 5510) = 5.4 *	0.52 *
Age x Sex	F(1, 5805) = 7.5 **	0.12 **
Income	F(1, 5416) = 30.7 ***	−0.84 ***
Income x Sex	F(1, 5416) = 4.1 *	0.45 *

*** *p* < 0.001, ** *p* < 0.01, * *p* < 0.05.
